# Bridging hard callus at 48 days in an open femoral shaft fracture with segmental defect treated with a first-stage Masquelet technique: I wasn’t expecting that

**DOI:** 10.1007/s11751-017-0300-z

**Published:** 2017-11-07

**Authors:** Andrew James Hotchen, Lynne V. Barr, Matija Krkovic

**Affiliations:** 1 Department of Trauma and Orthopaedic Surgery, Addenbrooke’s Hospital, Cambridge University Hospitals, Cambridge, CB2 0QQ UK; 2 Division of Trauma and Orthopaedic Surgery, University of Cambridge, Cambridge, CB2 0SP UK

**Keywords:** Masquelet procedure, Traumatic brain injury, Segmental defect

## Abstract

The Masquelet technique is a strategy for management of segmental bone defects. It is a two-stage procedure that involves inducing a synovial-like membrane that can be used for a bone graft. Segmental bone defects can occur following trauma and can accompany traumatic brain injury. There is a well-documented, albeit debated, association between traumatic brain injury and increased rate of new bone formation. Here, we present a case of unexpected callus formation in a segmental femoral fracture. The patient had a traumatic brain injury and was treated with the first stage of the Masquelet technique. Owing to the amount of large callus, a second stage of the Masquelet was not required. The patient recovered well from the injury and at 16-week follow-up was able to partially weight bear. A case similar to this has not previously been reported within the literature.

## Introduction

Acceleration of fracture healing has long been associated with traumatic brain injury (TBI) [1]. This has been supported by both murine studies [2] and clinical observation [3, 4]. Despite these studies, the actual mechanism through which this accelerated fracture healing occurs is yet to be elucidated. This new bone is thought to be either heterotopic ossification or new callus formation.

A segmental bone defect is one that extends across the width of the bone. These types of defect have a greater risk of non-union or infective complications [5]. One method of managing segmental bone defects is by using the membrane-inducing Masquelet technique. This is a two-stage technique that was described in 1986 which first uses a cement spacer to induce a synovial-like membrane [6, 7]. The membrane is composed of fibroblasts, myofibroblasts and collagen [8]. This biologically active membrane can be used as a chamber that improves vascularity and inhibits fibrous growth [7]. The cement space is removed and bone graft is inserted into this biologically active chamber during the second stage of the procedure.

Here, we report a case of unexpected copious callus formation in a segmental femoral fracture treated using the Masquelet technique in a patient with concomitant TBI.

## Case presentation

A previously fit and well 42-year-old male (with no history of hypertrophic ossifications) was admitted following a road traffic collision (RTC). At the scene of the accident, the patient had a Glasgow Coma Score (GCS) of 7 (eyes 2, verbal 2 and motor 3) and was intubated in a rapid sequence induction. He was then transferred to a Major Trauma Centre (MTC). The extent of the injuries included a right subdural haematoma with no mass effect, small subarachnoid haematoma, right open mid-distal femoral fracture, right proximal tibial fracture, right open proximal humeral fracture and right distal humeral fracture. Concomitant injuries included a small right pneumothorax, lung contusions, T1–4 spinous process fractures and a scapula fracture. An emergency intracranial pressure (ICP) triple bolt was inserted on admission, and the ICP was kept below 20 mmHg.

On admission, both distal humeral and femur were washed and debrided during the same operation. The femoral fracture was managed with a spanning external fixator, and a vacuum-assisted closure (VAC) device was applied. The distal humeral fracture was fixed with a 4-hole locking dynamic compression plate, also with a VAC dressing.

**Fig. 1:**
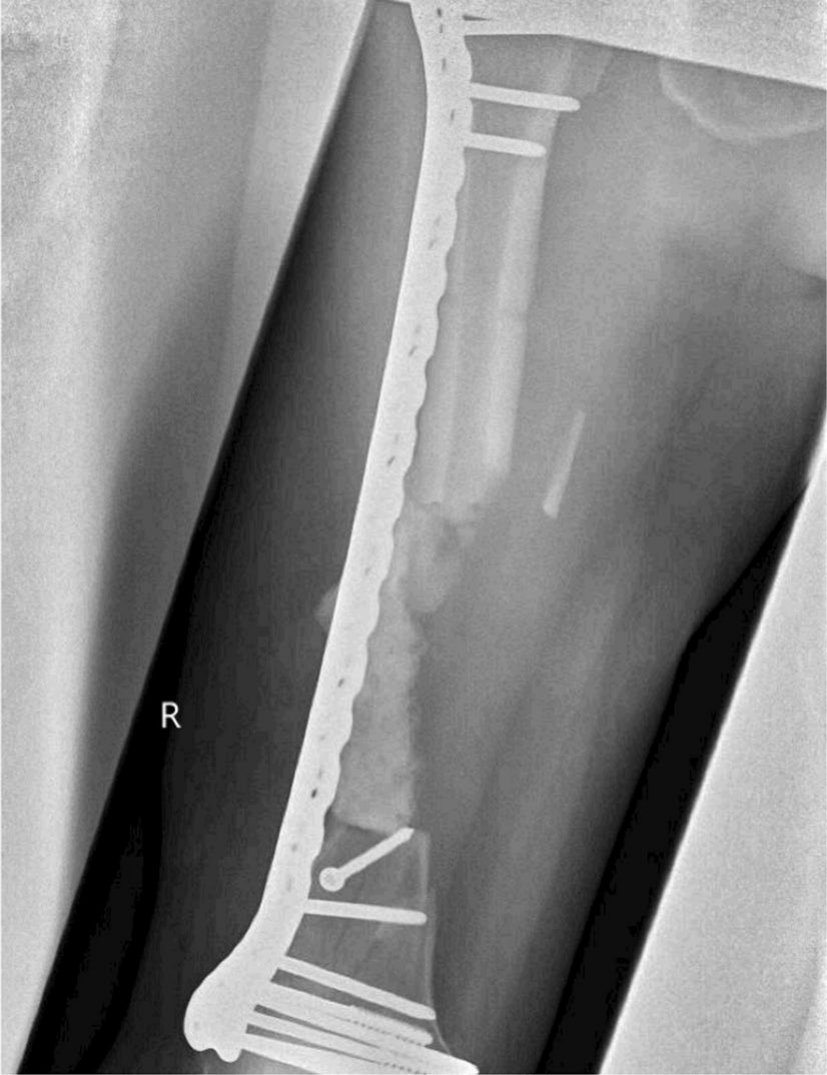
AP radiograph of femur showing the cement spacer and hard callus at the time of surgery

**Fig. 2:**
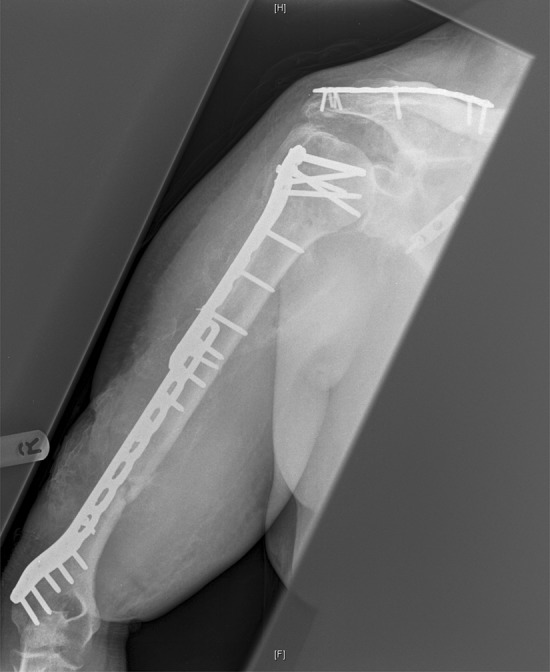
AP radiograph of the humeral fracture at 41 days after presentation

Definitive stabilisation was performed 6 days following admission for fractures. At this point, the first stage of the Masquelet procedure was performed for the femoral fracture. Following debridement, there was an 11-cm segmental defect within the mid-shaft of the femur. This was stabilised with an 18-hole lateral femoral plate with 2 partially threaded cancellous screws distally to compress the intercondylar split (Fig. 1). Palacos cement (Zimmer Biomet Holdings, inc., Warsaw, Indiana, USA), loaded with Stimulan beads containing gentamicin, was inserted into the segmental defect. Following the surgery, the patient was extubated and immediately regained a GCS of 13. This increased to a GCS of 15 5 h following extubation. The second stage of the Masquelet procedure was planned for and performed 48 days following the first stage. There were no procedures performed on the femur in between the two Masquelet stages, although five further washouts and one split skin graft was performed for the humeral fractures. Radiographs of the humeral fractures taken 41 days after presentation showed excessive heterotrophic ossification (Fig. 2).

On exposing the femur for removal of the cement spacer in the second stage of the Masquelet procedure, it was noted that hard callus had formed instead of the synovial-like membrane that was expected. In view of the almost circumferential new bridging hard callus that was present within the gap, this was not necessary. The new bone was gently elevated, the cement spacer removed, and the wound washed out with 0.9% sodium chloride. Stimulan beads with antibiotics were added, and the wound was closed. Following this operation, partial weight-bearing was advised.

**Fig. 3:**
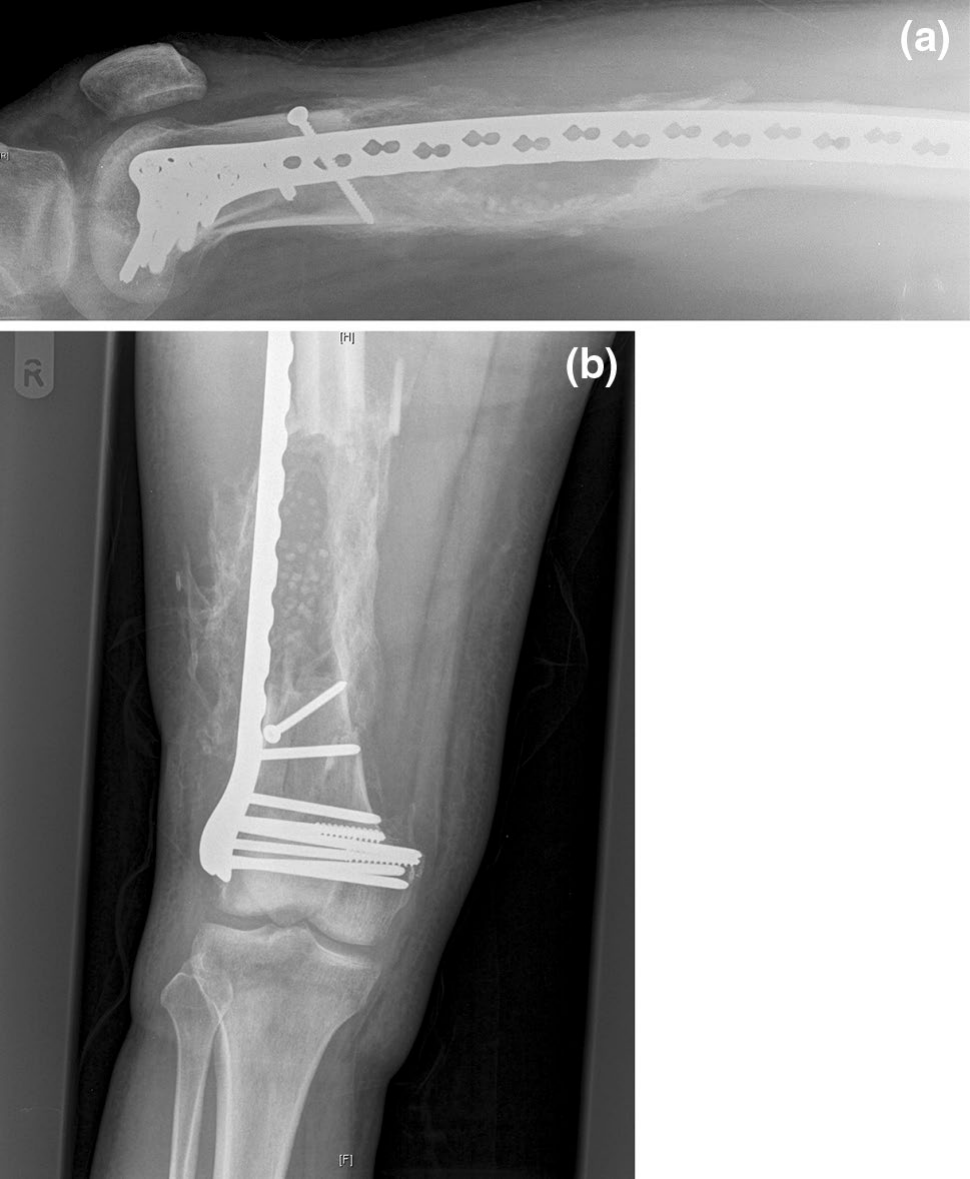
**a**, **b** AP and lateral of femur 35 days following the operation

**Fig. 4:**
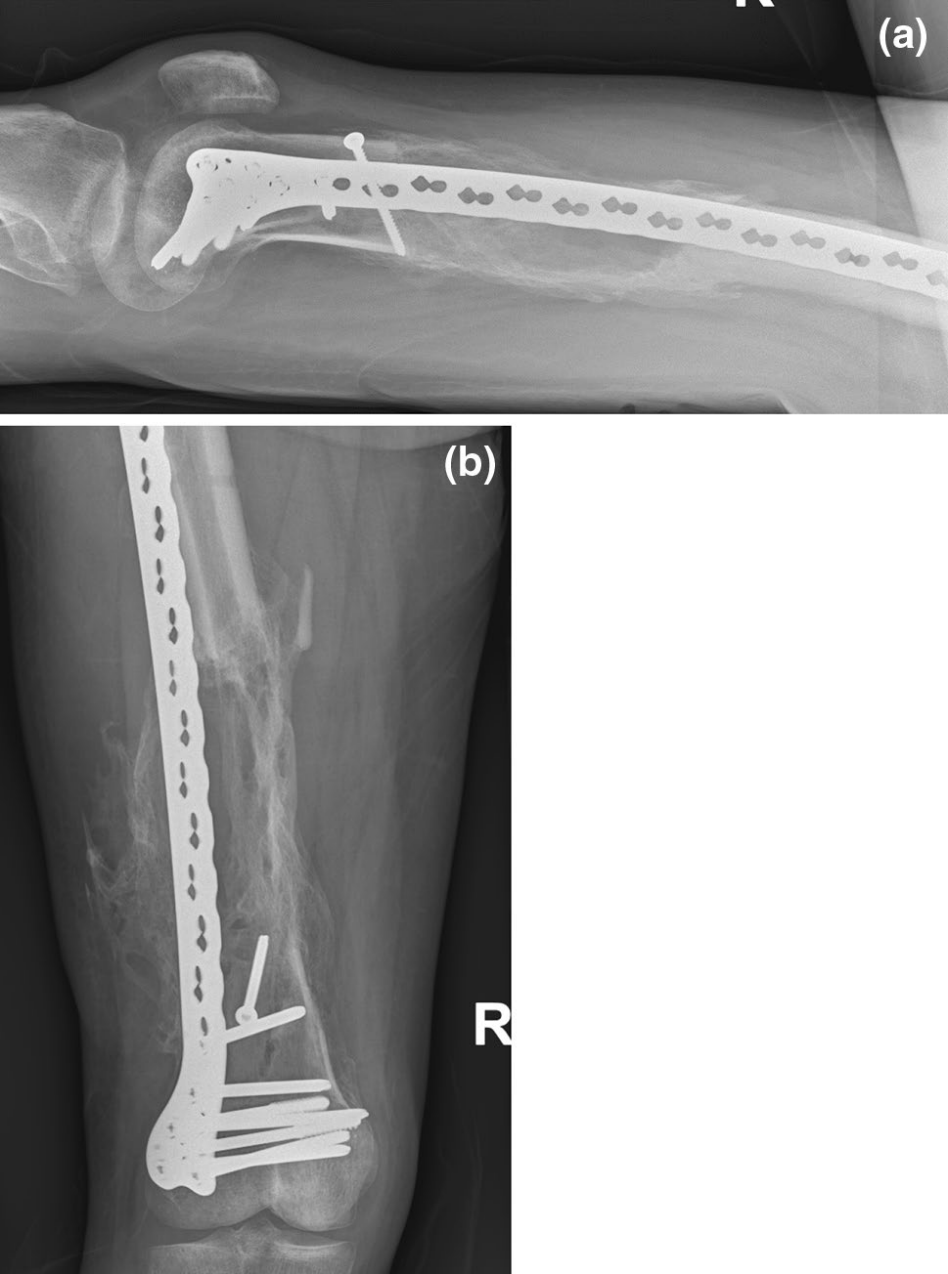
**a**, **b** AP and lateral of femur 70 days following the operation

The patient had an uncomplicated recovery and was discharged to a rehabilitation hospital 63 days following admission. Six weeks following discharge, the patient could bear full weight through the right leg. X-rays taken 35 and 70 days after surgery showed that callus was continuing to develop in the segmental defect and the fracture was beginning to unite (Figs. 3, 4). At most recent follow-up, 16 weeks following the surgery, the patient is mobilising well and not experiencing any discomfort in his right leg.

## Discussion

This case has presented a situation where, in the presence of TBI, there has been a rapid rate of hard callus formation following the Masquelet technique used for treating a large segmental femoral fracture. This callus, formed at 48 days after the injury, was enough to solidly bridge the 11-cm defect such that the bone transport was no longer indicated. The callus was left to consolidate the gap, and evidence of union segment was appearing on the most recent radiographs. There was an increased rate of callus formation than expected in this patient. This rapid rate of callus formation is likely attributable to the concomitant TBI sustained during the trauma.

The mechanism as to how TBI increases the rates of callus formation is not well understood. Callus is a feature of secondary (indirect) bone healing which commonly will occur from 5 days to 12 weeks after a fracture occurs. Cytokines, including tumour necrosis factor-α, interleukin-1 and interleukin-6, are released in the acute phase of the injury and are able to induce the formation of cartilaginous callus and promote angiogenesis [9]. Following TBI, these cytokines rise in both the serum and the cerebrospinal fluid [10, 11]. In addition to this, these cytokines can interact with mesenchymal stem cells (MSCs), attracting them to areas of damage. MSCs are integral in the regulation of fracture healing. They are able to respond to the micro-environment and adapt into differing cell types in addition to function as a signalling centre for other cells of the immune system [12]. These MSCs are also able to activate further immunological cascades, including a variety of bone morphogenetic proteins (BMPs), that enhance and activate bone growth in the periphery [13]. In rats, MSCs were able to proliferate at a significantly higher rate at the fracture site in those who had a TBI compared to those who did not [14]. It is not only mediators that are thought to increase the rate of callus formation in the presence of TBI. Leptin is another factor that is increased in the circulation following injury, specifically TBI. Leptin, which is also released in the stress response to cytokines and hormonal factors, has been shown to be positively correlated with amount of callus formation at a fracture site [15]. It is thought that the combination of these factors promotes the expedited callus formation in the TBI patient.

The Masquelet technique is a two-stage technique that uses a biologically active chamber for bone graft to encourage fracture union in segmental defects. In this case, there was callus formed within the fracture site that was stable enough to be used as a bridge between the two fracture ends.

The timescale of 48 days (6 weeks and 6 days) was long enough for the callus bridge to form. During this time, the patient underwent a number of surgical procedures and would have had a persistently elevated inflammatory marker level. This could have played a role in the continual recruitment of pro-osteogenic cytokines and factors.

## Summary

TBI-accompanying complex fracture patterns often present to Major Trauma Centres, and there is a long-standing association with an increased rate of callus formation. In this case, the rate of callus formation is dramatically increased from that expected, such that a second-stage Masquelet procedure was not necessary. To our knowledge, this phenomenon has not previously been reported in the literature.
